# Innovative sources for funding of viral hepatitis prevention and treatment in low- and middle-income countries: a roundtable meeting report

**DOI:** 10.1186/s41124-016-0022-8

**Published:** 2016-12-16

**Authors:** David FitzSimons, Greet Hendrickx, Johannes Hallauer, Heidi Larson, Daniel Lavanchy, Ina Lodewyckx, Daniel Shouval, John Ward, Pierre Van Damme

**Affiliations:** 1 305 Route des Alpes, 01280 Prévessin, France; 2 Viral Hepatitis Prevention Board (VHPB), Centre for the Evaluation of Vaccination, Vaccine & Infectious Diseases Institute, University of Antwerp, Universiteitsplein 1, B-2610 Antwerpen, Belgium; 3 Dietrich-Bonhoeffer-Klinikum, 17036 Neubrandenburg, Germany; 4 Department Global Health, University of Washington, Seattle, USA; 5 Department Infectious Disease Epidemiology, London School of Hygiene and Tropical Medicine, London, UK; 6 Ruelle des Chataigniers 1, CH-1026 Denges, VD Switzerland; 7 Institute of Gastroenterology and Hepatology, Liver Unit, Hadassah-Hebrew University Hospital, Jerusalem, 91120 Israel; 8 Division of Viral Hepatitis, Centers for Disease Control and Prevention, Atlanta, GA USA

**Keywords:** Viral hepatitis, Prevention, Control, Treatment, Funding, Low-and middle-income countries

## Abstract

Hepatitis B is preventable and hepatitis C is treatable even if still at a high cost; most people who are infected with hepatitis B or C virus have not been screened yet and are unaware of their infections; and most countries, especially developing countries, do not have a national plan to prevent and control viral hepatitis. The advent of effective new treatments for hepatitis C has been an agent of change, allowing consideration of the feasibility of eliminating that disease and accelerating the control of viral hepatitis generally. These facts inspired the Viral Hepatitis Prevention Board (VHPB) to organize a meeting in London (8–9 June 2015) on innovative sources for funding of viral hepatitis prevention and treatment in low- and middle-income countries. The main focus of the meeting was to provide an overview of current health systems controlling viral hepatitis in low- and middle-income countries (LMICs); to identify ways to increase political commitment and financial sustainability of viral hepatitis prevention and control programmes in such countries; to identify potential funders and explore new funding mechanisms; to discuss lessons learnt about funding other disease programmes; to investigate how to convince and motivate decision-makers to fund viral hepatitis programmes in LMICs; to provide options for improving access to affordable screening and treatment of viral hepatitis in LMICs; and to list the commitments required for funding by donors, including governments, bilateral and multilateral organizations, non-traditional donors, development banks, foundations, and commercial financial institutions.

To improve viral hepatitis prevention and treatment in LMICs participating hepatitis and financing experts identified the most urgent needs. Data on burden of disease must be improved. Comprehensive hepatitis policies and strategies should be drafted and implemented, and existing strategies and policies improved to increase access to treatment and prevention. Strong political will and leadership should be generated, potential partners identified and partnerships created. Potential funders and funding mechanisms have to be researched. The outcome of this meeting was integrated in a VHPB project to investigate creative financing solutions to expand access to and provision of screening and other preventive services, treatment and care of hepatitis B and C in LMICs. The report is available on www.vhpb.org.

The Viral Hepatitis Prevention Board (VHPB) organized a meeting in London (8–9 June 2015). Its purpose was four-fold. First it sought to examine how to increase financial sustainability of viral hepatitis prevention and control programmes and to raise political commitment to investing in viral hepatitis programmes in low- and middle-income countries (LMICs). Secondly, it would explore what lessons could be learnt from the funding of other disease programmes. Thirdly, it aimed to identify, or at least explore ways of identifying, potential funders and new funding mechanisms. Finally, it wanted to determine options for improving access to affordable screening and treatment of viral hepatitis in LMICs. The report reflects the presentations and discussions at the meeting and is therefore not a comprehensive analysis of the subject. It is recognized that viral hepatitis needs to be part of a broader, holistic and integrated approach, avoiding vertical programmes.

## Background

In 1992, the World Health Assembly (WHA) adopted resolution WHA45.17 on immunization and vaccine quality, urging Member States to introduce routine hepatitis B vaccination. More than 90% of the World Health Organization (WHO)’s 194 Member States have done so [1, 2]. According to WHO, investment in hepatitis B vaccination could prevent an estimated 4.8 million deaths between 2010 and 2020 in the 73 countries supported by the GAVI Alliance [3]. Large numbers of adults remain chronically infected with hepatitis B virus and are at risk of developing liver disease. For them, prolonged treatment is needed. Besides treatment, the major remaining challenges are to improve coverage of the birth dose of hepatitis B vaccine and to design good prevention and control strategies for viral hepatitis in LMICs.

For hepatitis C, the landscape is complex and rapidly changing. The epidemiology of, and responses to, hepatitis C are diverse. Large numbers of people infected with hepatitis C virus (HCV) are unaware of their infection. There is no vaccine, but recently licensed direct-acting antiviral agents (DAAs) are described as a “technological breakthrough”. They offer public health gains, with sustained viral response suggesting cure in more than 95% of cases within three months, and apparently little risk of resistance or relapse. Medically there is every reason to give treatment, but barriers remain, including access to care and significant costs. So far, discounts have been successfully negotiated in only a few countries and regions, generic formulations are being marketed, and tiered pricing is also expected to improve the situation as a result of more competition among pharmaceutical companies.

Although the subject of viral hepatitis prevention and control is receiving greater international attention, it faces not only declining funding for public health generally and an economic climate of austerity and shrinking budgets but also competition for funding and resources for other public health problems. UNAIDS, for instance, is facing a considerable funding gap. Governmental budgets are being squeezed, for example, by the high costs of medicines for cancer as well as the new DAAs for hepatitis C. Moreover, widespread introduction of DAAs creates other challenges, such as insufficient resources (human and financial), budgetary concerns about treating all people with chronic hepatitis C as well as the lack of solid data on the burden of disease, especially in LMICs. High quality tests to diagnose infection HCV and monitor response to therapy are widely available in most parts of the world, but they are not readily available or affordable in LMICs. At governmental and institutional levels, from the European Commission to governments of some LMICs, there is a lack of political will and financial investment. Viral hepatitis specifically and liver disease in general have been under-represented in health policies and funding.

In many countries the general population and even health professionals lack awareness about chronic viral hepatitis. Surveillance and screening are poor, and health systems’ capacity for preventive care and treatment is inadequate. Access to diagnostic and treatment services, where they exist, may be limited, with marginalized subjects facing further obstacles as well as stigmatization and discrimination. Approaches to control and prevention of viral hepatitis vary markedly between countries, and not many have developed realistic financial models to promote complete programmes for vaccination and disease elimination. Collaboration between governments and financial modellers might overcome the argument that lack of a financial model is a reason for not taking action.

## Burden of disease

The current burden of disease estimates indicate that 240 million people are chronically infected with hepatitis B virus, resulting in nearly 686,000 deaths each year from cirrhosis and liver cancer, and 130–150 million people are chronically infected with HCV, with around 700,000 deaths a year [4].

Although the incidence of hepatitis C is not increasing in many countries because of safer medical procedures and needle-exchange programmes, the burden of cirrhosis and liver cancer will continue to rise because of the long incubation of the disease. Mortality rates globally for cirrhosis and liver cancer have been increasing steadily for several years, reflecting infections acquired decades ago. Mathematical modelling shows that the burden of disease due to hepatitis C is likely to increase, rising to a peak around the year 2030. The overall burden of viral hepatitis, however, is underestimated because all viral causes of liver disease are not combined. Many estimates have under-represented the morbidity and mortality due to infection with HBV and HCV because cirrhosis and liver cancer were not included.. Besides rates, attention must be paid to absolute numbers; low rates of reported hepatitis cases in China and India hide the fact that millions of people are ill with viral hepatitis.

## Treatment

The new DAAs for treatment of hepatitis C have transformed prospects. According to some analyses they are cost-effective [5], but they are widely considered expensive (e.g. list price of about US$ 84,000 for treatment with sofosbuvir in the USA) and not necessarily affordable. One study shows that over the next five years the cost of treatment of all eligible patients in the USA would amount to US$ 65 billion, before offsetting for savings in care for hepatitis C [6]. These estimates must be considered low given that only half HCV-infected persons are diagnosed and even smaller proportions are in care settings where HCV treatment is provided [7]. Through mandated discounts for US government programmes and competition among pharmaceutical companies, the cost of treating HCV has declined. Recent studies also indicate that there is a wide variety in list price while net prices are lower and very similar between the US and other developed markets but even with discounted prices, significant budgetary impact from HCV treatments can be expected [8]. In the UK, National Health Service (NHS) England estimated that the cost of treating 20,000 people would reach £1 billion (not allowing for any negotiated discount) and recently announced that it would set aside more money for treatment with DAAs. The French Government negotiated deals under which a course of treatment with sofosbuvir will cost €41,000 (the cost for treating 200,000 infected people will amount to some €800 million) and treatment with the ledipasvir/sofosbuvir combination has been cut to €46,000. Altogether, about 15,000 people were treated in France in 2014.

Consideration of overall long-term medical costs will need to take account of savings in treatment of hepatitis C due to successful therapy with the new antivirals. Many patients in developed countries with end-stage liver disease, in many cases due to hepatitis C, need a liver transplant. Unless action is taken to control hepatitis C, expensive as it may be in the short-term, total long-term health care costs are likely to rise. At the European level, the European Liver Patients Association (ELPA) has used a model simulating the public budget impact of increasing treatment coverage with newest DAAs in France and Romania [9] to show that investing in the newest therapies is cost-effective, with short-term costs off-set in the longer run.

Participants stressed the need to perceive HCV as a community-acquired infection (rather than focusing on the concept of risk groups), with nosocomial and iatrogenic transmission important modes of transmission. In South Asia the reuse of syringes was highlighted as a major source of transmission (see for example [10]).

## Health systems in LMICs

Viral hepatitis should not be seen in a vacuum; strengthening health systems is high on the international public health agenda, including WHO’s programme of work and the 2030 Agenda for Sustainable Development. For viral hepatitis, like other public health diseases and conditions improvements are called for, from the training of medical students and health-care workers to screening and delivery of care and treatment services. LMICs need fully-costed strategies, policies and action plans (e.g. for testing, diagnosis, screening/surveillance, education, treatment and prevention) as well as training in health economics. The associated funding for implementation must be committed and sustainable. Surveillance and health information systems are also vital, with sufficient sensitivity to detect changing morbidity and mortality rates.

In some countries, political commitment and governmental leadership are evident, with written national plans and mainly State funding for programmes. In Pakistan, the Prime Minister took responsibility for a national prevention and control programme, and in Georgia the Government has introduced free treatment, held national workshops leading to national programmes, and collaborated with a pharmaceutical company. In other countries, the new DAAs are being introduced with donor support. Most countries do not have national plans and many lack surveillance and monitoring plans. A survey among WHO Member States notes that 37% countries have a plan and 29% have a governmental unit dedicated to hepatitis prevention and control [11]. The Pan American Health Organization (PAHO), however, is developing a regional plan of action for the prevention and control of viral hepatitis for the period 2016–2019 [12]. After the meeting^1^, the World Health Assembly adopted the global health sector strategy on HIV, viral hepatitis and sexually transmitted infections for the period 2016–2021 in resolution WHA69.22 in May 2016 [13]. Following this adoption the other WHO regions are executing or developing regional action plans. The viral hepatitis strategy considers that elimination of hepatitis B and C is feasible in the foreseeable future and sets out actions for Member States that take into account their national contexts and priorities as well as support and technical assistance for Member States.

Generally awareness of viral hepatitis is poor, and out-of-pocket payments (ranging from 10 to 80% in some countries) [14, 15] are necessary and pose a heavy burden on poor populations. In all countries priorities are distorted, with ad hoc resource allocations, cost-effective interventions not applied at scale, while spending is wasted on ineffective interventions despite scarce resources and inequitable access driven by vested interests. In LMICs, the capacity for priority-setting is limited.

Overall, the list of main obstacles to prevention and control of viral hepatitis, and indeed effective, strong and sustained health systems, is long. Challenges identified included funding (for testing and treatment) and the disparity in funding between viral hepatitis and diseases with higher profiles; shortage and lack of training of health-care workers; low levels of knowledge and awareness about viral hepatitis; poor infrastructure; weak data; inadequate infection control (including massive overuse of injections); inadequate supplies of equipment; lack of licensing of non-medical facilities; and difficulties in controlling the quality of services in private sector.

## Who are some of the main players?

### Intergovernmental level

After years of neglect of viral hepatitis there are encouraging signs of movement: the disease is moving up towards its deserved place on the international public health agenda. At the same time, LMICs have to cope with emerging communicable diseases and threats such as Ebola, Zika and dengue, not to mention malaria and tuberculosis, and expanding epidemics of noncommunicable diseases, including obesity, as well as humanitarian crises and emergencies. At the intergovernmental level, “combat viral hepatitis” is included in target 3.3 of the United Nations’ Sustainable Development Goal on health. The use of the verb “combat” dismays many hepatitis professionals, given the use of the term “eliminate” for other diseases, especially as vaccination offers the possibility of preventing hepatitis A and B and treatments are available to cure hepatitis C.

WHO has drafted a global strategy to eliminate viral hepatitis as a public health concern and has regional plans in place; it is also supporting the development of some national plans^2^ [13]. Besides its regional plan, PAHO has its existing Revolving Funds and Strategic Fund, which offer models for introducing vaccines. WHO’s Regional Office for Europe is preparing to implement the European Vaccine Action Plan 2016–2020, which includes a regional goal for controlling hepatitis B virus infection.

### International and national levels

Partners such as the European Centres for Disease Prevention and Control (ECDC) and the United States Centers for Disease Control and Prevention (CDC) are active. For example, CDC has issued guidelines for testing for chronic hepatitis B and recently recommended that everybody in the USA born between 1945 and 1965 be screened for hepatitis C. CDC’s collaboration with the Government of the Republic of Georgia on a project to eliminate hepatitis C could be an example for other LMICs. Civil society bodies such as the World Hepatitis Alliance have been instrumental in generating pressure on governments and international organizations as well as delivering solid research.

### National governments

Some national governments have acted strongly to tackle viral hepatitis. Brazil and Egypt have been particularly active at the intergovernmental level. Georgia has a State programme of targeted treatment, a population-based prevalence survey, and a five-year strategy and action plan [16]. Mongolia, through its prevention, control and elimination programme, aims to halve deaths due to liver cancer and cirrhosis by 2020 [17, 18]. Egypt, which has the highest reported prevalence rate of hepatitis C in the world, introduced a national plan in 2013 and in 2015 negotiated very favourable terms for the supply of treatments for hepatitis C [19–21]. France has a national strategy and plan for prevention and control, with a designated unit responsible for coordination and implementation, and, to the extent that the outcomes of negotiations are in the public domain, has been very successful in Europe in lowering the price of treatment of hepatitis C, with further discounts if certain volume targets are met [22, 23]. Other countries (Italy, Portugal, Spain and Switzerland) have also negotiated discounts but the resulting prices have not been made public.

### Civil society

*The Gavi, the Vaccine Alliance*, a global public-private partnership, set the standard for accelerating the introduction of new and underused vaccines in many LMICs. Its support also led to a massive drop in the price of the pentavalent vaccine for the poorest countries over the course of a decade. The Alliance has further goals for strengthening the capacity of health systems, improving the predictability and sustainability of financing for immunization, and shaping markets in order to lower and sustain the prices of vaccines. These goals, its vision to see the connection between childhood health and future economic prospects, its learning-by-doing approach, and its proven success since its inception in 2000 provide models for obtaining and sustaining lower prices for the treatment of viral hepatitis. On the other hand, its ability to negotiate lower prices was predicated on advance purchase commitments, eligibility for its support was limited in terms of a country’s income, and it relied on substantial donor and private-sector funding. Extrapolation of the model to viral hepatitis is not a given and would need careful consideration and modifications.

*UNITAID* is a global health initiative, launched by the governments of five countries in 2006 as a non-profit organization partially financed by a small levy on airline tickets and part of one country’s tax on CO_2_ emissions from air travel. Its main focus has been on HIV/AIDS, tuberculosis and malaria, but its strategic objectives for 2013–2016 include increasing access to treatment for co-infections with HBV and HCV. It disburses about US$ 200 million a year in grants. Through its mandate to work on co-infections, and focusing on the product rather than the disease, it does not pay for treatment cost but it is supporting WHO’s programme to prequalify medicines. In 2015, UNITAID’s Executive Board adopted a resolution [24] supporting its need to focus strategically on the development of better tools to diagnose HCV infection, in particular in cases of HIV/HCV co-infection.

Another major player in the international public health scene is the *Global Fund to Fight AIDS, Tuberculosis and Malaria*. It annually disburses about US$ 4 billion but faces numerous demands on its budget in the context of its three priority diseases [25, 26]. Despite cogent arguments about the similarities in disease burden of viral hepatitis compared to that of HIV/AIDS and the wide disparities in funding, some of its donors are said to be urging it to resist extending its mandate to cover viral hepatitis. Having supported much work on diagnostics, the Global Fund described UNITAID as a key partner and trail-blazer, in particular through working to shape markets for new health products. By providing evidence of success through demonstration projects, UNITAID could make it feasible for the Global Fund to act on the recent opening for supporting HIV/HCV co-infections.

In various countries, both civil society and the pharmaceutical industry have teamed with governments to introduce prevention and control programmes. For example, the *Médecins sans Frontières*’ Access Campaign has begun screening and treatment programmes for hepatitis B and C in the Democratic Republic of the Congo, India (Manipur province), Myanmar and Pakistan. The *Clinton Health Access Initiative* is working with health ministries in several countries, such as Myanmar and Rwanda, to support the introduction of sustainable government-led programmes for treating hepatitis C. The company *MSD India* initiated a programme to educate patients and their families about hepatitis C and to help to manage the cost of treatment; its Project Sambhav was launched in the Punjab in 2012 with two elements: disease management and micro-financing. The *Onom Foundation* in Mongolia, working closely with the national Government, has instituted a prevention campaign that includes prevention, screening and early diagnosis, on-site rapid testing projects, lobbying, and the creation of a national database on viral hepatitis.

The *International Decision Support Initiative* was created by the UK’s National Institute for Health and Care Excellence, with support from the UK Government, the Bill & Melinda Gates Foundation and the Rockefeller Foundation, in order to guide decision-makers in LMICs to effective and efficient resource allocation strategies for improving people’s health.

Researching existing and potential partners for projects and programmes to prevent, control and eventually eliminate viral hepatitis as a public health problem, including mechanisms such as technology transfer and local production (see below) is still a long way off.

## Innovative and potential new funding mechanisms

Some current funding mechanisms and initiatives have been extremely successful. For instance, the Gavi, the Vaccine Alliance created innovative financing mechanisms such as the International Finance Facility for Immunisation, which raises funds by issuing bonds in the capital markets [27]. It was also the midwife to advance market commitments that help the development and manufacture of new and better vaccines for use in LMICs by guaranteeing a market, reducing unpredictability or volatility of demand, and increasing competition and innovation between companies and organizations [28–30]. Advance market commitments greatly accelerated the introduction of pneumococcal conjugate vaccine and have the potential to dramatically accelerate the introduction of new vaccines and technologies into LMICs. The PAHO Revolving Fund for Vaccine Procurement, which pools the resources of 41 countries and territories, has enabled the procurement of supplies at the lowest prices for the whole Region of the Americas.

The principle of financing UNITAID through a levy is now established, even though it continues to rely on a small number of contributors. Its success is held up as a justification for introducing innovative financing mechanisms. Indeed, UNITAID commissioned a study that showed that a levy on financial transactions could be implemented and tax thereon collected.

Integrating health care financing or non-financial health care services (e.g. health education) into a micro-financing institution can be an opportunity to soften financial risks associated with poor health [31]. *MSD’s microfinancing scheme* in India used a financial partner to provide zero-interest, collateral-free loans to eligible patients to pay for their medicines over a period of time. It took many months to overcome the difficulties of adapting the credit company’s criteria and patient profiles, introducing IT systems and getting rural customers used to modern financial services. Yet, the scheme is reported to be working. Given its very low default rate, it might be expanded.

Health insurance is seen as a possible pathway to pursue universal health coverage, reducing out-of-pocket expenditures and improving access to care and financial protection in LMICs, and several health insurance projects have been successfully introduced [32, 33]. Many countries are using health insurance as a path towards universal health coverage, but it is a partial solution not a panacea. A lesson from the HIV/AIDS experience is that disease-specific insurance does not work. Inclusive insurance is proposed as a means of focusing on the target population. Given that most health costs are covered in LMICs by out-of-pocket payments, inclusive insurance mobilizes resources, pools risks and can promote healthy behaviours, early detection and treatment. Private and public health insurance programmes can be complementary but need good design and oversight in order to avoid fragmentation and excessive costs. Partnerships are crucial. *Abt Associates’* International Health Division is testing plans to introduce coverage for viral hepatitis.

A community-based alternative is the rural health cooperative. Worldwide, about one billion people are members of cooperatives, and more than 100 million work in them. Health cooperatives deliver their members in rural areas medical care that is not available through public or private health programmes [34]. In 2003, China launched the New Rural Cooperative Medical System, a system of mutual assistance for health protection through risk pooling. The structure is managed, organized and subsidized by the central, provincial and county governments. It incorporates two major principles: voluntary participation by the rural population and emphasis on protection against catastrophic illnesses [35].

Other potential mechanisms include social/development investment bonds for health. Private finance initiatives have been largely used to improve infrastructure, but their performance has generated a poor public image. Apart from the bonds of the *International Finance Facility for Immunisation*, bonds have generally not been applied for health improvement largely because of the lack so far of accurate data on disease burdens and epidemiology and the inability to monitor changes in outcome. With advances in these areas, it is now possible to consider a bond or loan (depending on fiscal environment) to finance preventive health, linking governments, health-care providers and financial institutions for piloting in different policy settings. So far, interest in such schemes has been predominantly in Anglo-Saxon societies.

Many companies have departments for corporate social responsibility with whom innovative partnerships could be forged [15, 36]; for viral hepatitis an example of an approach could be that a pharmaceutical company foundation subsidizes the cost of hepatitis medication for members of a national health insurance scheme, under which hepatitis prevention and treatment protocols are tested while offered as covered services. The partners learn together about disease burden and how to identify/treat patients cost-effectively, although the cost-effectiveness analyses would need to take into consideration the possibility of re-infection after successful treatment [37, 38]. There could be a sliding scale for price of medicines based on volume, providing a shared incentive to bring a programme to scale.

A continuing theme was the considerable scope for increasing partnerships and multisectoral cooperation. In 2010, the World Health Assembly passed a resolution calling on countries to “constructively engage the private sector in providing essential health-care services” [39, 40]. Numerous partnerships have sprung up at country and lower levels between private sector (in particular the pharmaceutical industry) and governments or health care centres to finance screening and treatment. These activities tend to be scattered and uncoordinated.

New and innovative funding is an active field of study, not just for viral hepatitis. WHO is holding a financing dialogue with its Member States and key non-State contributors, including a focus on sustainable financing and coordination to meet priority health needs generally in developing countries. Overall, however, donor funding for health is becoming increasingly scarce; add the need for sustainable and predictable funding and the task becomes even harder. Identifying the success factors and obstacles for these various mechanisms would contribute to the better definition of new funding mechanisms, as envisaged by VHPB and the International Federation of Pharmaceutical Manufacturers & Associations (IFPMA) [15]. Key factors already identified include: political will (including the will to innovate); targeting with a tight focus (with clearly defined inputs and measurable outcomes); and effectiveness in both financial and medical terms of reference.

## Issues and needs

The dramatic more than five-fold growth in financing for health that began in 1991 has plateaued since 2010 [41]. Impressive gains have been made in health; these need to be maintained, yet evidence suggests that the priority of health is in decline. *The Lancet* concluded that the outcome of the third Financing for Development conference, held in 2015, demonstrated that “health is no longer a priority” [42]. Health accounts for only one of the 17 Sustainable Development Goals. There are concerns that the level of the next replenishment of the Global Fund to Fight AIDS, Tuberculosis and Malaria might not match that of previous rounds, and the organization is being urged to focus more on health systems strengthening.

To pay for health, countries in receipt of official development assistance are increasingly being asked to generate domestic funding [43]. For viral hepatitis, the potential number of cases for prevention and treatment is enormous, yet the area is only slowly being recognized as a funding priority, although in most countries its financing will face an uphill struggle against established and new competitors for funding as well as constrained resources. While attention currently focuses on hepatitis C, hepatitis A, B, D and E must not be overlooked.

Health-care systems are seen as black holes for resources, while priorities for using scarce resources are often distorted and access to services is inequitable. However, it seems to be less a question of whether a country or society can pay for expensive treatments or vaccines than whether they can successfully negotiate discounts, alone or collaboratively. Then the issue becomes one of affordability rather than cost. Recent moves in government funding of treatment of hepatitis C suggest that decisions are increasingly being based on affordability rather than just cost-effectiveness and budget impact.

Much has been made in public health of the value of cost-effectiveness analyses, and indeed they can de-politicize issues. Although cost-effectiveness is a very important criterion, it is not the only one for hepatitis C treatment programmes, even with forecasts of cure rates of up to 95% within 12 weeks. Indeed, budget impact analysis and immediate availability of funds and resources are the keys to initiate sustainable programmes. The social costs besides medical costs need much better quantification.

The keystone of successful negotiations on the price of treatments or vaccines for the prevention and control of viral hepatitis is the existence of a robust national plan and strategy. Countries that have such plans and strategies have negotiated the lowest prices for treatments of hepatitis C.

To make a future free of viral hepatitis feasible and affordable [44], the clear message was that strategies are needed to break the vicious circle of which action comes first. There will not be one solution, but rather a suite of approaches. At a time when public health policies are shifting away from vertical programmes, is there a misperception of viral hepatitis programmes as such a programme? One overarching solution will be to include viral hepatitis in comprehensive universal health plans rather than disease-specific plans. To reach WHO’s goals, treatment and testing must be part of a comprehensive national hepatitis plan, in addition to vaccination, surveillance, blood safety, infection control, harm reduction, and sexual health that are also needed to achieve dramatic reductions in transmission and disease.

Lowering the price of medicines is important but not the only solution. The introduction of new DAAs underlines the need for priority setting and the resolution of the medical, ethical and political issues about who to treat, when to treat, what messages to convey to people infected with HCV, and the rationing of treatment because of cost.

In addition to affordable treatments and sufficient budgets, simplifying, and thereby reducing the cost of, other elements in the spectrum of care and systems, such as diagnostic tests, will strengthen prevention and control. The Gavi, the Vaccine Alliance’s experience provides an example of such an approach. Besides provision of the materials for viral hepatitis care and treatment services, the fundamental questions are how to improve access, how to use existing staff and services to deliver good-quality care, and how to incentivize countries to invest in screening programmes.

Other issues arise with communication. Messages using language and terminology that are coherent and consistent will avoid misperceptions, stigmatization and potential loss of credibility; for example, “elimination”, “eradication”, “affordable”, “reasonableness”, “value” and “fairness” need definition in the context of viral hepatitis and public health. In addition, it is vital to tailor language to specific audiences and target groups.

With the growing recognition globally of the importance of viral hepatitis in the public health agenda, it is a time to capitalize on enthusiasm and opportunity. Many clinical trials of different combinations of therapies are under way and being reported. Publications on the subject abound, including guidelines for care and treatment and many studies of cost-effectiveness. Numerous meetings have been held, including a global hepatitis summit in Glasgow in 2015. New DAAs and combinations are being licensed and in some cases they are also being launched in developing countries before developed countries.

## Some lessons learnt from HIV/AIDS

What is happening with the prices of medicines and access to care, the beginnings of activism and the partnerships between governments, the health sector, industry and financial institutions echoes the early days of AIDS, but at an accelerated rate.

Political will and commitment, donor support and community acceptance are crucial to efforts to raise awareness, attract funding and implement projects or programmes. Advocacy needs to be maintained, especially to keep viral hepatitis high on the political agenda; if possible, champions should be identified – whether individuals or countries (as is happening now, as witnessed by the activities of Brazil, Egypt, Slovenia and France). Leverage of the private sector is also a key factor. As with AIDS, viral hepatitis cuts across many disciplines, from tax, economics and international trade to ethics, politics and health care policy – sectors offering sources of expertise and potential partners.

Commitment and financing will follow only when there is a unified strategy at the political and technical levels, coupled with continued preparatory work and a coalition of the willing, driven by a sense of urgency. Services for viral hepatitis should be integrated into other health delivery services. Optimism and perseverance are valuable and necessary qualities in order to ensure continued momentum.

The initiative launched by UNAIDS and WHO in 2003 to target treating three million people infected with HIV in LMICs with antiretroviral medicines highlighted the value of demonstration projects and realistic scale up. The equivalent initiative for viral hepatitis now is to diagnose 90% of people with chronic hepatitis, to treat 90% of those diagnosed, and to treat and cure 90% of that group. The AIDS experience has paved the way for introduction of new hepatitis C treatments into developing countries, and we are already seeing the pressure resulting from introduction of generic medicines and competition.

Not everything learnt from HIV/AIDS can be successfully replicated or adapted. For instance, a “risk-group focus” is inappropriate and unhelpful; activism and advocacy by celebrities are not likely to have the same impact as in the AIDS era, − even though the same anger is there among those without access to treatment.

## Conclusions

### The way forward

AIDS has its International AIDS Society to coordinate academic and research aspects; an equivalent body for viral hepatitis could perform a similar function. The International Decision Support Initiative could be approached to serve as a platform for engagement of the pharmaceutical sector and other stakeholders.

The business case for funding prevention and treatment of viral hepatitis needs to be fully researched, developed and strongly made. Potential traditional funders and new funding mechanisms need to be vigorously and thoroughly investigated and persuaded to support prevention and control plans.

More pilot and demonstration projects should be launched. Successful models (e.g. microfinancing) need to be analysed to determine the reasons for their success and the likelihood of their being able to be transferred or adapted to other settings. Focusing on health technology assessment could serve as a tool to help priority setting for resource allocation.

Updated policies and guidelines will be of added value to screening, treatment, care and disease management. Infection control and injection safety need urgently to be improved in some countries. And the focus on prevention as treatment needs to be maintained.

A further step forward could be the creation of an alliance or fund for the development of infrastructure for viral hepatitis prevention and control, formulation of national plans and introduction of treatment, and/or the creation of an initial non-institutional or informal group to undertake such work, as in the lead up to the formation of the Gavi, the Vaccine Alliance. This is a possible role for stakeholders such as VHPB and IFPMA. Such a body could also aim to avoid duplication and provide coherence to the public health approaches through coordination of parties – from research and work on strategy development to the organizing of further meetings of interested parties.

In summary, recommended actions highlighted during the meeting include:

- better definition of the burden of disease and socio-economic costs, together with analysis of data and trends; improved quality of data
- formulation of comprehensive policies and strategies for prevention and control of viral hepatitis at national level, with clear definition of objectives and priority setting
- generation of commitment and political will through continued advocacy, and identification of strong leadership
- creation of an alliance or fund for the development of infrastructure for viral hepatitis prevention and control, formulation of national plans and introduction of treatment, and/or the creation of an initial non-institutional or informal group to undertake such work
- identification of a broad base of potential partners in an alliance or coalition to advance and coordinate activities on prevention and control of viral hepatitis at an international level
- research into identifying the best practices and the success factors of (demonstration) projects, programmes, financing mechanisms (including private sector funding through grants, loans or bonds) and activities that improves the treatment and prevention accessibility, and stimulate further development

## Abbreviations

CDC: Centers for Disease Control and Prevention
DAAs: Direct-acting antiviral agents
ECDC: European Centres for Disease Prevention and Control
ELPA: European Liver Patients Association
HCV: Hepatitis C virus
IFPMA: International Federation of Pharmaceutical Manufacturers & Associations
LMICs: Low- and middle-income countries
PAHO: Pan American Health Organization
VHPB: Viral Hepatitis Prevention Board
WHA: World Health Assembly
WHO: World Health Organization

## Footnotes

1 A positive event following the meeting was the launch of a national policy on viral hepatitis by the Federal Nigerian Government. National prevalence rates of hepatitis B and C are estimated to be 11% and 2.2% respectively. The “Prevent Hepatitis – Act Now” programme reflects the fact that most of the predisposing health risks for viral hepatitis are avoidable through adequate awareness, behavioural change and screening of blood for transfusion. Measures will include increased vaccination against hepatitis B, safe injection programmes, expanded screening and treatment services, harm reduction and moves to prevent stigmatization and discrimination. It will be essential for adequate domestic funding to be assured [45].

2 Adopted after the meeting.
